# The Effects of Intraoperative Remifentanil Infusion on Postoperative Opioid Consumption in Patients Who Underwent Total Knee Arthroplasty with Femoral Nerve Block

**DOI:** 10.3390/jcm12154975

**Published:** 2023-07-28

**Authors:** Chanjong Chung, Jinyoung Choi, Taeyoung Lee, Sangyoong Park

**Affiliations:** Department of Anesthesiology and Pain Medicine, Dong-A University Hostpital, 26 Daesingongwon-ro, Seo-gu, Busan 49201, Republic of Korea; cjchung@dau.ac.kr (C.C.); cjy821@naver.com (J.C.); pinpd@dau.ac.kr (T.L.)

**Keywords:** analgesics, anesthesia, femoral nerve block, hyperalgesia, opioid, postoperative pain, remifentanil, total knee arthroplasty

## Abstract

(1) Background: Remifentanil is used for intraoperative pain control; however, it has several side effects, such as hypotension and opioid-induced hyperalgesia. We aimed to determine whether an intraoperative remifentanil infusion may increase postoperative opioid consumption in patients undergoing total knee arthroscopy (TKA) under femoral nerve block (FNB) in addition to general anesthesia. (2) Methods: We randomly assigned 66 patients who underwent total knee arthroplasty to the remifentanil (R) and control (C) groups. All patients underwent FNB and popliteal artery and posterior capsule of the knee (iPACK) block in addition to sevoflurane-based general anesthesia. Postoperative pain control was achieved using intravenous patient-controlled analgesia (IV-PCA) fentanyl. We recorded IV-PCA fentanyl consumption at various postoperative timepoints, numerical rating scale (NRS) scores, intraoperative changes in vital signs and index of nociception (qNOX), ephedrine consumption, postoperative side effects, satisfaction, and sleep quality. (3) Results: The primary outcome (the cumulative IV-PCA fentanyl usage within 48 h postoperatively) was significantly lower in the C group (541.1 ± 294.5 µg) than in the R group (717.5 ± 224.0 µg) (*p* < 0.001). The secondary outcome (the cumulative IV-PCA fentanyl usage within 12, 24, and 72 h) was lower in the C group than in the R group and the mean arterial pressure was lower in the R group than in the C group from immediately after tourniquet on to immediately after tourniquet off. The heart rate was lower in the R group from immediately after incision to immediately after irrigation. There was no significant between-group difference in the perioperative qNOX and NRS scores at rest and activity except for NRS scores at 72 h postoperatively. Ephedrine use was higher in the R group than in the C group (*p* = 0.003). There was no significant between-group difference in the incidence of postoperative nausea and vomiting, nor in the postoperative satisfaction and sleep quality. (4) Conclusions: Avoiding intraoperative remifentanil infusion may reduce total opioid consumption in patients undergoing FNB before TKA.

## 1. Introduction

Opioid use continues to be a significant burden in Korea with respect to side effects and morbidity [1,2]. Opioid use has dramatically increased over the past decade [3], especially for noncancerous pain [4,5]; accordingly, there has been a concomitant increase in opioid-related deaths and overdoses [3,6]. Therefore, continuous efforts have been made to reduce the use of opioid analgesics.

Surgery is among the risk factors for opioid use [7,8,9]. Continuous intravenous infusion of remifentanil is widely used for intraoperative pain control since it has fast onset and offset. However, opioid analgesics may lead to opioid-induced hyperalgesia (OIH), which may increase the usage of opioid analgesics for postoperative pain control, resulting in opioid-related side effects [10,11,12,13]. While remifentanil may improve hemodynamic stability, it may also lead to hypotension and bradycardia.

Total knee arthroplasty (TKA) is a common surgical treatment for patients with osteoarthritis and rheumatoid arthritis. However, it often involves severe postoperative pain, which can significantly affect patient rehabilitation and satisfaction. Therefore, pain control is considered a significant factor in TKA and is generally controlled using opioid analgesics and nerve blocks [14,15]. However, excessive opioid use can cause side effects, including nausea, vomiting, and difficulty breathing in the recovery room or ward; moreover, opioid dependence can easily develop.

Since the femoral nerve is a crucial nerve for pain control after TKA, femoral nerve block (FNB) is among the most commonly used nerve blocks in TKA [15]. However, since FNB can only control pain in the front of the knee, interspace between the popliteal artery and posterior capsule of the knee (iPACK) block can be additionally used to control pain behind the knee [16,17,18].

We hypothesized that intraoperative remifentanil infusion could increase postoperative opioid consumption and pain due to OIH and avoiding remifentanil infusion could maintain hemodynamic stability and the index of nociception (qNOX), reduce numerical rating scale (NRS) score, and minimize the side effects of remifentanil. Therefore, this study aimed to evaluate the effects of intraoperative remifentanil infusion on postoperative opioid consumption in patients who underwent TKA with FNB.

## 2. Materials and Methods

### 2.1. Study Design

This randomized study was approved by the Research Ethics Committee of the hospital and registered prior to patient recruitment with the Clinical Research Information Service at CRIS.NIH.go.kr (Accessed on 13 April 2022). This study was conducted and reported in accordance with the principles of the Declaration of Helsinki and the Consolidated Standards for Reporting Trials (CONSORT). This study was conducted after checking the medical records, confirming the patient’s medical history, and explaining the study to the patient.

### 2.2. Study Population

The inclusion criteria were as follows: age of 30–90 years, American Society of Anesthesiologists physical status classification of I–III, and patients scheduled for TKA under general anesthesia at our hospital from April to November 2022. The exclusion criteria were as follows: infection at the block site, chronic opioid dependence, neurological deficits, hypersensitivity to remifentanil (Hana Pharmaceutical, Seoul, Republic of Korea) or ropivacaine (Hanlim Pharmaceutical, Seoul, Republic of Korea), uncontrolled diabetes, psychosocial problems, left ventricular ejection fraction < 0.35, morbid obesity (body mass index > 35 kg/m^2^), pregnancy, inability to understand the use of intravenous patient-controlled analgesia (IV-PCA) fentanyl, and refusal to participate. All patients provided informed consent and were instructed on the use of IV-PCA fentanyl and the NRS. An NRS score of 0 represents no pain while a score of 10 represents the highest pain level that can be perceived.

### 2.3. Randomization and Blinding Method

The patients were assigned to the remifentanil (R) group or control (C) group through randomization using a computer program (Excel software, Microsoft, Redmond, WA, USA). The group allocation was blinded using concealed envelopes and identified only by the nurse preparing for anesthesia. In the R group, in addition to routine anesthesia care, attending anesthesia providers adjusted the remifentanil infusion to maintain hemodynamic stability. Routine anesthesia care was provided in the C group. Neither the subjects, caretakers, nor investigators involved in postoperative patient management or data collection were aware of the group assignment.

### 2.4. Interventions

All patients fasted from midnight and received glycopyrrolate 0.004 mg/kg via intramuscular injection and famotidine 20 mg intravenously before entering the operating room. After entering the operating room, the blood pressure, heart rate (HR), and qNOX [19,20] levels were measured to establish the baseline under standard monitoring. Both groups underwent an ultrasound-guided peripheral nerve block (PNB) before anesthesia. Both the FNB and iPACK blocks were performed with the patient placed in the supine position. A high-frequency linear ultrasound transducer (3–16 MHz; HS40 device; Samsung Medison Ultrasound, Seoul, Republic of Korea) was transversely placed into the inguinal crease. The femoral nerve, which is located adjacent to the femoral artery, typically appears as a hyperechoic or ovoid structure. It is superficially located in the iliopsoas muscle group and deeply in the fascia lata and iliaca. A needle tip (a 22-gauge Echoplex Plus needle; Vygon Vet. Ltd., Swindon, UK) was placed toward the bottom of the fascia iliaca using an in-plane approach. After negative aspiration, FNB was performed using 20 mL of 0.375% ropivacaine. The iPACK block was performed using the same probe until the identification of the femoral shaft and popliteal vessel in the popliteal crease or proximal area. A needle was placed in the space between the popliteal artery and femur in the anteromedial-to-posterolateral direction using an in-plane technique. After negative aspiration, the iPACK block was performed using 20 mL of 0.375% ropivacaine. After performing the block, the onset of the sensory block was assessed by applying the jagged edges of a broken tongue depressor to the skin within the sensory distribution of the femoral nerves. This assessment included the anterior and medial aspect of the thigh, medial aspect of the lower leg, medial aspect of the knee, and the big toe. The pinprick test was conducted at 2 min intervals up to a maximum of 30 min. If not all of the femoral nerve supply sensations were blocked during this time, the outcome was considered a failure to block.

### 2.5. Intraoperative Management

Propofol was controlled at 1–2.5 mg/kg according to the patient’s weight and age. Additionally, after confirmation of loss of consciousness, 0.6–0.9 mg/kg of rocuronium was used as a muscle relaxant. Subsequently, tracheal intubation was performed, followed by the administration of sevoflurane (1–3 vol%) and monitoring of Conox (Fresenius Kabi, Bad Homburg, Germany) to adjust the index of consciousness (qCON) and qNOX to 40–60. qCON and qNOX are defined as a 0–99 dimensionless score based on the analysis of EEG data. As the scale value increases, it means recovery of consciousness and great pain, respectively. After intubation, ventilation was initiated at a 40% fraction of inspired oxygen and 3 L/min flow.

The only between-group difference was the intraoperative use of remifentanil. In the R group, anesthesia was maintained with 2 mg/40 mL remifentanil at the target concentration of the effect site (Ce) of 2–6 ng/mL using a target-controlled infusion pump (Agilia, Minto model effect site; Fresenius Kabi). An anesthesiologist adjusted the Ce to maintain hemodynamic stability.

The preoperatively collected vital signs were set as the baseline values. Moreover, in case of a change in the intraoperative value by >20%, systolic blood pressure < 85 mmHg, mean arterial pressure (MAP) < 65 mmHg, or HR < 45 beats per min persisting for > 5 min, ephedrine was administered and recorded. Additionally, the MAP, HR, and qNOX were recorded at the following time points: T0 = preoperative period, T1 = immediately after tourniquet on, T2 = immediately after incision, T3 = immediately after bone cutting, T4 = immediately after implant insertion, T5 = immediately after irrigation, T6 = immediately after tourniquet off, and T7 = in the recovery room. All management procedures under general anesthesia were performed by an anesthesiologist who was not involved in the postoperative protocol.

### 2.6. Postoperative Management

Postoperative pain was controlled using an IV-PCA fentanyl pump (Accuuser Plus M1015M; Woo Young Medical, Seoul, Republic of Korea). Specifically, 20 µg/kg of fentanyl and 0.6 mg of ramosetron were mixed with 0.9% normal saline until a total volume of 100 mL was obtained. The infusion rate, bolus dose, and lockout time were set at 0.6 mL/h, 0.8 mL, and 10 min, respectively. Rescue analgesics (50 mg of tramadol) were intravenously injected up to thrice per day, as per the patient’s request (if the NRS score was greater than 4 points). Tramadol was converted and integrated using an intravenous fentanyl equivalent (Tramadol 10 mg to be equivalent to fentanyl 10 µg). An intravenous injection of 0.3 mg of ramosetron was administered if the patient complained of postoperative nausea and vomiting (PONV). After the surgery, a cushion was placed under the patient’s knee for elevation and rest at a 45° angle.

### 2.7. Outcome Measurement

The primary outcome was the cumulative IV-PCA fentanyl usage within 48 h postoperatively [21]. The secondary outcomes were cumulative IV-PCA fentanyl usage at 6, 12, 24, and 72 h postoperatively; NRS score (at rest) at 6, 12, 24, 48 and 72 h postoperatively; NRS score (at activity) at 24, 48, and 72 h postoperatively; change in vital signs and qNOX; ephedrine consumption; postoperative side effects; satisfaction; and sleep quality. Vital signs and qNOX changes were measured over eight periods (T0 to T7). Additionally, ephedrine consumption based on hypotension and bradycardia was recorded. Preoperative NRS scores were obtained before the block. The NRS score at rest reflected the pain experienced by the patient during the resting state. The NRS score at activity reflected the pain experienced at the start of postoperative rehabilitation using a continuous passive motion machine (FLEXIEE 2.1; Caretech, Seongnam, Republic of Korea), which was performed daily (twice a day for 30 min per session, at a 45° angle) starting from 24 h postoperatively, increasing in the painless range. Postoperative side effects were recorded. Nausea scores for PONV (0, no nausea; 1, mild nausea; 2, severe nausea; 3, severe nausea and vomiting) were used, and a nausea score of 1 or greater was considered to be PONV. Sleep quality was recorded at 24 h postoperatively, while patient satisfaction and intention to block were recorded using a questionnaire at 72 h postoperatively.

### 2.8. Statistical Analysis

The primary endpoint was cumulative fentanyl consumption within 48 h postoperatively. Joly et al. [21] reported an increase in the 48 h postoperative cumulative opioid consumption of 86 (59–109) mg when using remifentanil. Assuming that a 50% decrease in fentanyl consumption is clinically significant, each group required 27 patients to detect a difference with a type Ⅰ error of 0.05 and a power of 0.8. To account for a 20% dropout rate, 33 patients were recruited in each group.

Variables were summarized by frequency and percentage for categorical data and by mean ± standard deviation and median (range) for numeric data. Group differences were tested using the chi-squared test or Fisher’s exact test for categorical data and the independent *t*-test or Mann–Whitney U test for numeric data, as appropriate. The normality of distribution was assessed using the Shapiro–Wilk test.

Given the nature of repeated measurements, model fitting was performed using a generalized linear mixed model (GLMM) with random intercepts. The GLMM model comprised repeated measures of numeric variables as dependent variables; group, time, and group × time interaction as fixed effects; and subject as a random effect. To avoid making any assumptions regarding the covariance structure, we used an unstructured covariance matrix that was allowed to differ across the groups for the GLMM analysis.

All statistical analyses were performed using the statistical software IBM SPSS Statistics for Windows (version 26.0; IBM Corp, Armonk, NY, USA) and R statistical software (version 3.4.0; R Foundation, Vienna, Austria, http://www.r-project.org/, accesed on 1 January 2023). Statistical significance was set at *p* < 0.05.

## 3. Results

**Figure 1:**
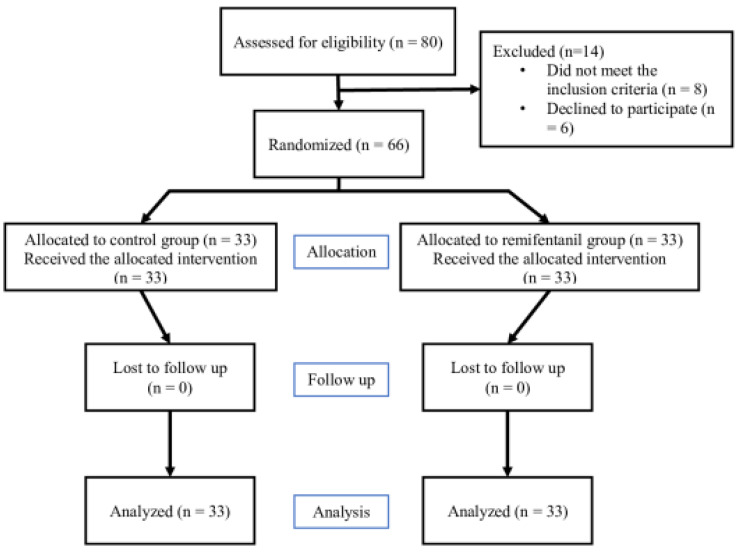
CONSORT diagram.

From April to November 2022, six out of eighty patients were rejected; moreover, eight patients were excluded based on the inclusion/exclusion criteria. Finally, 66 patients met the study criteria, with 33 patients being enrolled in each group (Figure 1).

There was no significant between-group difference in the basic characteristics (Table 1).

**Figure 2:**
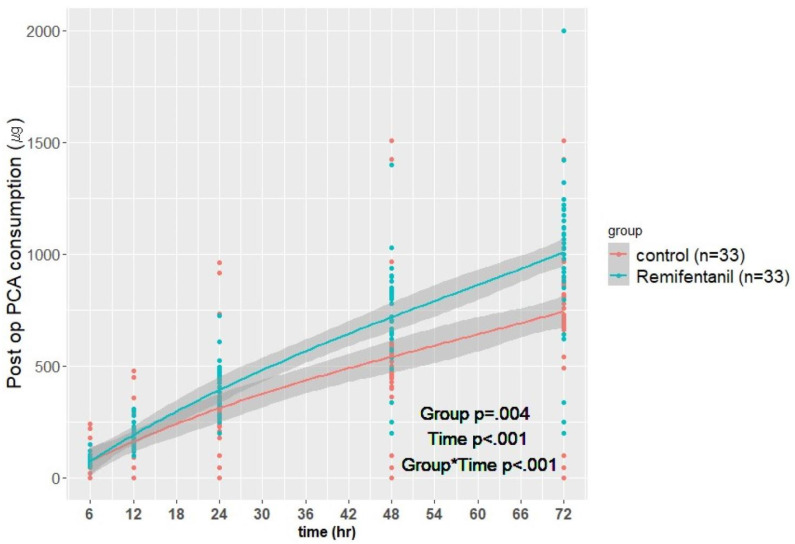
Postoperative (post op) intravenous patient-controlled analgesia (IV-PCA) consumption at 6 (*p* = 0.019), 12, 24, 48, and 72 h (all *p* < 0.05).

Figure 2 and Table 2 show the postoperative IV-PCA fentanyl consumption. The cumulative IV-PCA fentanyl consumption within 48 h postoperatively was 717.5 ± 224.0 μg and 541.1 ± 294.5 μg in the R and C groups, respectively (*p* < 0.001). Moreover, total IV-PCA fentanyl consumption at 6, 12, 24, and 72 h postoperatively was higher in the R group than in the C group (*p* = 0.019, *p* < 0.001, *p* < 0.001, and *p* < 0.001, respectively). There was no significant between-group difference in the NRS score at rest and at activity; however, the NRS score was higher in the R group than in the C group at 72 h postoperatively (*p* = 0.033, *p* = 0.017, respectively; Table 2).

**Figure 3:**
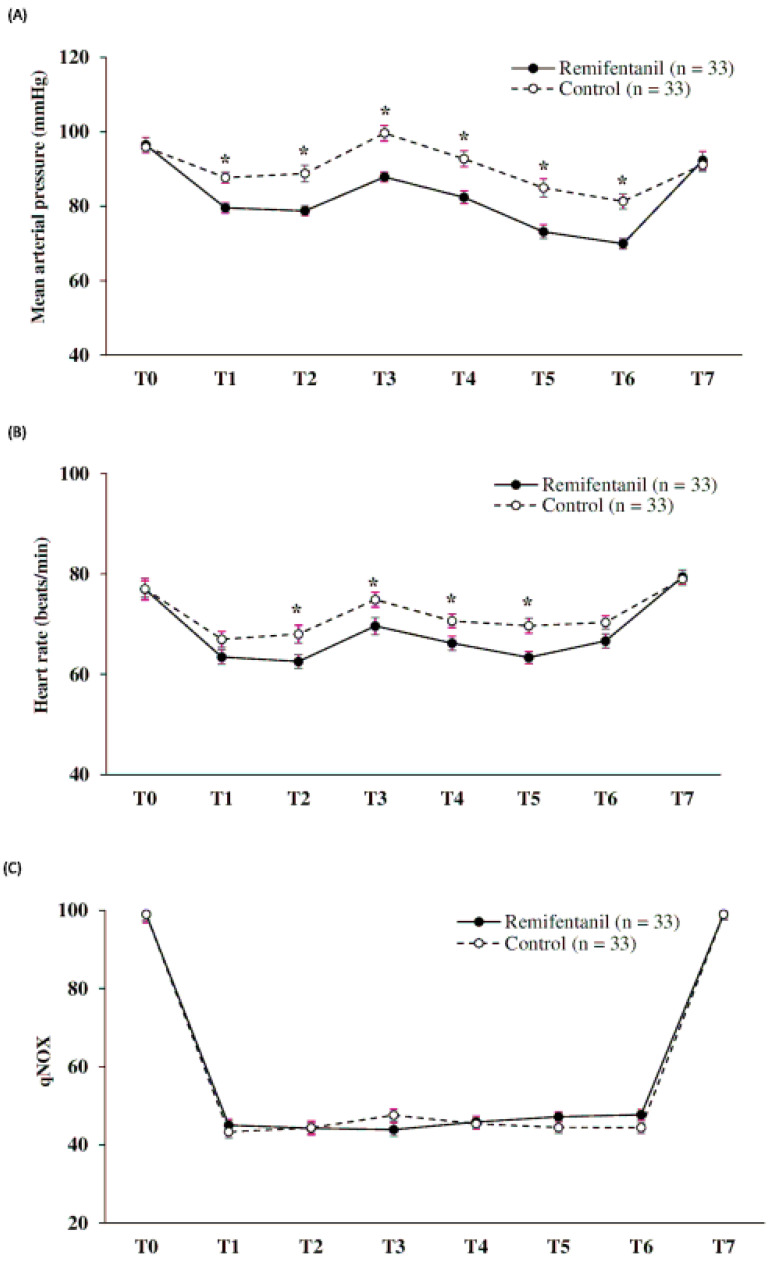
Intraoperative outcomes: (**A**) Mean arterial pressure; T1–T6 (all * *p* < 0.05). (**B**) Heart rate; T2–T5 (* *p* = 0.01, * *p* = 0.025, * *p* = 0.029, * *p* = 0.002, respectively). (**C**) Index of nociception (qNOX);T0 = at preoperation, T1 = immediately after torniquet on, T2 = immediately after incision, T3 = immediately after bone cutting, T4 = immediately after implant insertion, T5 = immediately after irrigation, T6 = immediately after torniquet off, and T7 = in the recovery room.

Figure 3 shows the intraoperative MAP, HR, and qNOX outcomes. MAP was lower in the R group than in the C group in all periods except for T0 and T7 (all *p* < 0.001). Although there was no significant between-group difference, the HR was lower in the R group from T2 to T5 (*p* = 0.011, *p* = 0.025, *p* = 0.029, and *p* = 0.002, respectively). There was no between-group difference in the qNOX levels. The amount of ephedrine used for perioperative hypotension was higher in the R group than in the C group (13.0 ± 11.3 mg vs. 5.2 ± 7.1 mg, *p* = 0.003; Table 3).

The postoperative outcomes are presented in Table 3. Rescue tramadol use in the R group (42.4%) was higher than that in the C group (21.2%); however, this difference was not statistically significant (*p* = 0.064). Finally, there was no between-group difference in the incidence of PONV nor in postoperative satisfaction or sleep quality.

## 4. Discussion

The purpose of this study was to compare postoperative IV-PCA fentanyl use with or without remifentanil infusion in patients undergoing TKA with FNB to determine the effect of remifentanil on the amount of opioid analgesics used postoperatively.

Regarding the primary study outcome, the total IV-PCA fentanyl consumption at 48 h postoperatively was higher in the R group by 32.6% compared with that in the C group. Additionally, the difference gradually increased from 6 h to 72 h postoperatively (Figure 2, Table 2). This suggests that intraoperative remifentanil use may increase postoperative opioid use, which indicates a possible occurrence of OIH. Severe pain may occur following orthopedic surgery performed with PNBs, especially within the first 24–48 h. Generally, pain lasts for 72 h; however, PNBs can yield analgesic effects for up to 24 h, which can reduce opioid usage [22,23,24]. Therefore, this could suggest the IV-PCA fentanyl consumption difference increased after the PNB effect decreased.

Remifentanil is prophylactically administered to patients at risk of postoperative pain since it is reliable and allows for rapid recovery [13]. Since it does not accumulate, unlike other opioid analgesics, it is not significantly affected by the injection time. Accordingly, it is more hemodynamically stable than other opioid analgesics; moreover, continuous intravenous infusion of remifentanil is recommended to reduce the variability of anesthesia and depth of analgesia. However, patients who received remifentanil intraoperatively required more postoperative analgesics, which could be attributed to OIH. Compared with other opioid analgesics, remifentanil more frequently causes OIH [10].

Additionally, remifentanil infusion at the Ce has been shown to affect OIH [12,13,21,25,26]. OIH is characterized by signaling pathway hypersensitivity due to the use of opioid analgesics, which is followed by hypersensitivity to pain stimuli and a greater requirement for opioid analgesics. Although the lasting effects of OIH remain unclear, they can range from 30 min to ≥24 h or even up to 3 months [12,13,27]. Therefore, in this study, we monitored IV-PCA consumption up to 72 h postoperatively. The NRS score was also significantly higher in the R group at 72 h postoperatively. These findings are suggested to be attributed to persistent OIH.

Even though there was no between-group difference in the NRS score for 48 h postoperatively, IV-PCA fentanyl consumption in the R group was higher than that in the C group starting from 6 h postoperatively (Figure 2, Table 2). Thus, we could assume that the NRS score did not increase due to the higher IV-PCA fentanyl consumption.

Previous studies have shown that patients did not respond to intraoperative stimuli when qNOX levels were well controlled [19,20,28]. In a study of 60 patients who underwent general anesthesia with propofol and remifentanil, Jensen et al. demonstrated that qNOX can detect subtle changes in nociceptive balance by showing a series of significant correlations. In our study, there was no significant between-group difference in qNOX levels (Figure 3C). This indicated that qNOX was well controlled by PNB alone, i.e., without remifentanil, and that anesthesia was properly achieved, as demonstrated by the absence of response to intraoperative stimuli.

Inconsistent with previous studies [29,30], compared with those of the C group, the R group showed a higher occurrence of hypotension and perioperative ephedrine consumption (Table 3). This finding suggests that intraoperative hemodynamic instability may result from remifentanil infusion for intraoperative pain control.

This study has several limitations. First, both groups showed moderate pain, with an NRS score ≥ 4 after surgery. If the pain was caused by OIH, it should be addressed through other multimodal regimens, such as non-steroidal anti-inflammatory drugs (NSAIDs), rather than opioids. Second, some patients could have experienced rebound pain. When the effect of the PNB is diminished, the pain rapidly increases, which is termed rebound pain [5,31,32]. Therefore, effectively managing rebound pain has become an important concern in pain control [33]. However, we used the iPACK block in addition to FNB. Moreover, the NRS score of the C group was highest at 12 h postoperatively, which suggests the possibility that rebound pain had appeared at this time point [22,23,24], which suggested that pain was aggravated by the additional block. However, single-infusion PNB is currently considered an integral part of multimodal analgesia for postoperative pain, which comprises acetaminophen, NSAIDs, and opioids. PNB is safe and provides effective analgesia for 6–8 h, which contributes to reducing opioid analgesia requirements and PONV, as well as accelerating hospital discharge [34]. Third, there was a long time interval for the assessment of postoperative IV-PCA fentanyl usage. IV-PCA fentanyl consumption was monitored only at 24 h intervals from 24 h postoperatively, so we could not determine the changes between assessment points. Accordingly, subtle changes could have been observed if the time interval had been shorter.

## 5. Conclusions

In conclusion, in patients undergoing TKA after PNB, sevoflurane-based general anesthesia with remifentanil can increase the amount of opioids required for postoperative pain control. Therefore, without intraoperative remifentanil, we could reduce total opioid consumption during and after TKA, maintaining qNOX values and hemodynamic stability, as well as minimizing side effects.

## Figures and Tables

**Table 1:** Patient demographics and surgical data.

		Group		
Variable	Overall (*n* = 66)	Remifentanil (*n* = 33)	Control (*n* = 33)	*p*
**History**				
Osteoarthritis	55 (83.3)	27 (81.8)	28 (84.8)	0.741 *
Rheumatoid arthritis	11 (16.7)	6 (18.2)	5 (15.2)	
**Age (yr)**	71.3 ± 7.2	71.7 ± 6.7	70.8 ± 7.7	0.525 ^†^
**Gender**				
Male	13 (19.7)	6 (18.2)	7 (21.2)	0.757 *
Female	53 (80.3)	27 (81.8)	26 (78.8)	
**Height (cm)**	154.6 ± 6.8	154.0 ± 6.9	155.1 ± 6.7	0.525 ^†^
**Weight (kg)**	63.0 ± 8.7	62.6 ± 8.2	63.3 ± 9.3	0.737 ^‡^
**BMI (kg/m^2^)**	26.2 ± 2.9	26.2 ± 2.6	26.3 ± 3.2	0.937 ^‡^
**ASA class**				
1	0 (0.0)	0 (0.0)	0 (0.0)	0.786 *
2	47 (71.2)	24 (72.7)	23 (69.7)	
3	19 (28.8)	9 (27.3)	10 (30.3)	
**Surgical site**				
Right	35 (53.0)	19 (57.6)	16 (48.5)	0.459 *
Left	31 (47.0)	14 (42.4)	17 (51.5)	
**Surgery time (min)**	118.3 ± 11.3	116.8 ± 12.3	119.9 ± 10.1	0.264 ^‡^
**Anesthesia time (min)**	175.3 ± 14.5	174.9 ± 17.0	175.7 ± 11.7	0.814 ^‡^
**Blood loss (ml)**	325 ± 130	329 ± 131	321 ± 130	0.814 ^‡^
**Preoperative pain score (NRS)**	2.6 ± 1.1	2.6 ± 1.2	2.6 ± 1.1	0.989 ^†^
**Remifentanil total use (µg)**	1161.8 ± 206.7	1161.8 ± 206.7	-	

Data are presented as mean ± standard deviation or number (%), unless otherwise indicated. ASA (American Society of Anesthesiologists); BMI (body mass index); NRS (numerical rating scale). * *p* values were derived from the chi-square test. ^†^ *p* values were derived from the Mann–Whitney U test. ^‡^ *p* values were derived from independent *t*-test. The Shapiro–Wilk test was employed for test of normality assumption.

**Table 2:** Between-group comparison of changes in the postoperative IV-PCA consumption, pain score at rest, and pain score at activity.

	Group			Analysis for Repeated Measures	
Variable	Remifentanil (*n* = 33)	Control (*n* = 33)	*p* *	Source	*p* ^†^
**Postoperative IV-PCA consumption (µg)**					
6 h	79.3 ± 18.4	77.6 ± 47.9	0.019	Group	0.004
12 h	183.9 ± 55.7	155.8 ± 99.5	<0.001	Time	<0.001
24 h	398.6 ± 108.5	316.5 ± 199.3	<0.001	Group × Time	<0.001
48 h	717.5 ± 224.0	541.1 ± 294.5	<0.001		
72 h	1008.1 ± 339.0	744.1 ± 298.0	<0.001		
**Pain score at rest (NRS)**					
6 h	3.2 ± 1.7	3.9 ± 1.8	0.121	Group	0.026
12 h	3.9 ± 1.8	4.3 ± 1.9	0.440	Time	0.077
24 h	4.2 ± 1.6	4.1 ± 1.6	0.528	Group × Time	0.013
48 h	4.2 ± 1.1	4.1 ± 1.7	0.343		
72 h	4.5 ± 1.5	3.8 ± 1.7	0.033		
**Pain score at activity (NRS)**					
24 h	4.9 ± 1.4	4.8 ± 1.7	0.434	Group	0.031
48 h	4.9 ± 1.0	4.7 ± 1.8	0.257	Time	0.382
72 h	5.1 ± 1.4	4.3 ± 1.8	0.017	Group × Time	0.012

Data are presented as mean ± standard deviation or number (%), unless otherwise indicated. IV-PCA (intravenous patient-controlled analgesia); NRS (numerical rating scale); * *p* values were derived from the Mann–Whitney U test. ^†^ *p*-values were derived from the generalized linear mixed model. The Shapiro–Wilk test was employed for test of normality assumption.

**Table 3:** Intraoperative and postoperative outcomes.

	Group		
Variable	Remifentanil (*n* = 33)	Control (*n* = 33)	*p*
**Rescue tramadol use**			
Yes	14 (42.4)	7 (21.2)	0.064 *
No	19 (57.6)	26 (78.8)	
**Perioperative ephedrine (mg)**	13.0 ± 11.3	5.2 ± 7.1	0.003 ^†^
**Nausea or vomiting**			
Yes	11 (33.3)	11 (33.3)	1.000 ^‡^
No	22 (66.7)	22 (66.7)	
**Quality of sleep at 24 h**			
Poor	1 (3.0)	1 (3.0)	0.708 ^‡^
Fair	30 (90.9)	27 (81.8)	
Good	2 (6.1)	5 (15.2)	
**Satisfaction score (0–100) at 72 h**	76.2 ± 7.5	77.6 ± 10.0	0.280 ^†^
**Would undergo the block again (*n*) at 72 h**			
Yes	32 (97.0)	32 (97.0)	1.000 ^‡^
No	-	-	
Ambivalent	1 (3.0)	1 (3.0)	

Data are presented as mean ± standard deviation or number (%), unless otherwise indicated. * *p* values were derived from the chi-square test. ^†^ *p* values were derived from the Mann–Whitney U test. ^‡^ *p* values were derived from the Fisher’s exact test. The Shapiro–Wilk test was employed for test of normality assumption.

## Data Availability

The data presented in this study are available on reasonable request from the corresponding author.
